# Temperature and time stability of whole blood lactate: implications for feasibility of pre-hospital measurement

**DOI:** 10.1186/1756-0500-4-169

**Published:** 2011-05-30

**Authors:** Christopher W Seymour, David Carlbom, Colin R Cooke, Timothy R Watkins, Eileen M Bulger, Thomas D Rea, Geoffrey S Baird

**Affiliations:** 1 Division of Pulmonary and Critical Care Medicine, Harborview Medical Center, Seattle, WA, USA; 2 KL2 Clinical Research Scholar, University of Washington Institute of Translational Health Sciences, Seattle, WA, USA; 3 Division of Pulmonary and Critical Care Medicine, University of Michigan, Ann Arbor, MI, USA; 4 Robert Wood Johnson Foundation Clinical Scholars Program, University of Michigan, Ann Arbor, MI, USA; 5 Department of Surgery, Harborview Medical Center, University of Washington, Seattle, WA, USA; 6 King County Medic One, Division of General Internal Medicine, University of Washington, Seattle, WA, USA; 7 Department of Laboratory Medicine, University of Washington, Seattle, WA, USA

## Abstract

**Background:**

To determine the time and temperature stability of whole blood lactate using experimental conditions applicable to the out-of-hospital environment.

**Findings:**

We performed a prospective, clinical laboratory-based study at an academic hospital. Whole blood lactate was obtained by venipuncture from five post-prandial, resting subjects. Blood was stored in lithium heparinized vacutainers in three temperature conditions: 1) room temperature (20°C), 2) wrapped in a portable, instant ice pack (0°C), or 3) wet ice (0°C). Lactate concentrations (mmol/L) were measured at 0, 5, 10, 20, and 30 minutes after sampling, and compared using repeated measures analysis of variance.

Mean baseline lactate among resting subjects (N = 5) was 1.24 mmol/L (95%CI: 0.49,1.98 mmol/L). After 30 minutes, lactate concentration increased, on average, by 0.08 mmol/L (95%CI: 0.02,0.13 mmol/L), 0.18 mmol/L (95%CI: 0.07,0.28 mmol/L), and 0.36 mmol/L (95%CI: 0.24,0.47 mmol/L) when stored in wet ice, ice pack, and room temperature, respectively. The increase in lactate was similar in samples wrapped in portable ice pack or stored in wet ice at all time points (p > 0.05), and met criteria for equivalence at 30 minutes. However, lactate measurements from whole blood stored at room temperature were significantly greater, on average, than wet ice or portable ice pack within five and ten minutes, respectively (p < 0.05).

**Conclusions:**

Whole blood lactate measurements using samples stored in a portable ice pack are similar to wet ice for up to 30 minutes. These conditions are applicable to the out-of-hospital environment, and should inform future studies of pre-hospital measurement of lactate.

## Background

Early recognition of critical illness may lead to improved risk stratification, prognostication, and application of life-saving treatment. Practitioners often use blood lactate, an easily-measured biomarker in hospital emergency care settings, to identify critically ill patients at greater risk for organ dysfunction, occult hypo-perfusion, and mortality. Lactate concentrations may also inform treatment decisions, such as the initiation of early-goal directed therapy in septic shock or traumatic injury [1,2].

For both injured and non-injured patients, early recognition and triage of critically ill persons occurs before hospital arrival. Out-of-hospital triage models that identify patients at greatest risk for critical illness may be accurate, but require additional sophistication to reduce under and over triage [3]. To this end, out-of-hospital biomarkers, such as pre-hospital lactate, offer a potential method to improve discrimination [4]. During emergency medical services (EMS) care, blood lactate could be measured from whole blood specimens obtained during venipuncture which are transported to hospital laboratories or from handheld, portable devices [5]. Handheld devices for point-of-care (POC) lactate or arterial blood gas analysis are validated in hospital-based settings [5], and are a potential triage tool in the EMS care of the critically ill. Yet, this approach is not standard because of logistical, cost, and effectiveness considerations. One concern is the time-dependent stability of the sample. Venous samples may be readily available after intravenous catheterization by EMS, yet little data guides post-sampling conditions during EMS transport. Future comparative studies of POC lactate may also be informed by a referent standard using venous whole blood. These studies are challenging due to: 1) variability in out-of-hospital intravenous catheterization and venous sampling rates, 2) extended transport times of whole blood samples to central laboratories (> 15 minutes), and 3) well-recognized, pre-analytical instability of whole blood lactate during room-temperature storage or transport [6].

In this study, we sought to determine the temperature and time stability of venous, whole blood lactate measurements in healthy volunteers using both time intervals and storage methods applicable to the out-of-hospital environment.

## Materials and methods

We conducted a prospective, laboratory-based cohort study in five healthy volunteers. We obtained whole blood in three 5 mL lithium heparin vacutainer tubes (Becton Dickinson, Franklin Lakes, New Jersey) from five seated, postprandial volunteers through antecubital fossa venipuncture. We measured whole blood lactate (mmol/L) from each tube on the ABL800 blood gas instrument (Radiometer, Westlake, OH) immediately, and at 5, 10, 20, and 30 minutes after the initial measurement on each tube. The tubes were maintained at different temperatures after the initial measurement: 1) 20°C - room temperature 2) 0°C-wrapped in portable ice pack (Instant Cold Pack, Kimberly-Clark, Roswell, GA), and 3) 0°C-wet ice (referent condition).

We evaluated differences in mean lactate concentration across time and temperature conditions with repeated measures, two-way analysis of variance (ANOVA). We used Bonferroni post-tests to determine if pairwise differences reached statistical significance (p < 0.05). Mean change in lactate values at each time point are plotted with 95% confidence interval for each temperature condition. We decided *a priori *that a clinically significant difference in lactate is 0.2 mmol/L [6]. Because failing to reject the null hypothesis does not imply equivalence, [7] we evaluated the confidence interval for the difference in lactate increase (from baseline) for wet ice and ice pack at 30 minutes. We considered the difference in the increase in lactate concentration not clinically significant if the 95% confidence interval for the difference fell entirely between 0 and 0.2 mmol/L.

Using the measured assay coefficient of variation of 2%, we estimated that a sample size of five patients was required to detect a difference between 4 and 4.1 mmol/L (the minimum change detected by the instrument) assuming alpha = 0.05 and beta = 0.3. This study was approved by the University of Washington Institutional Review Board as part of an ongoing quality assurance project in the clinical laboratory. All data was analyzed using Prism 5.0 (GraphPad, La Jolla, CA).

## Results

**Figure 1:**
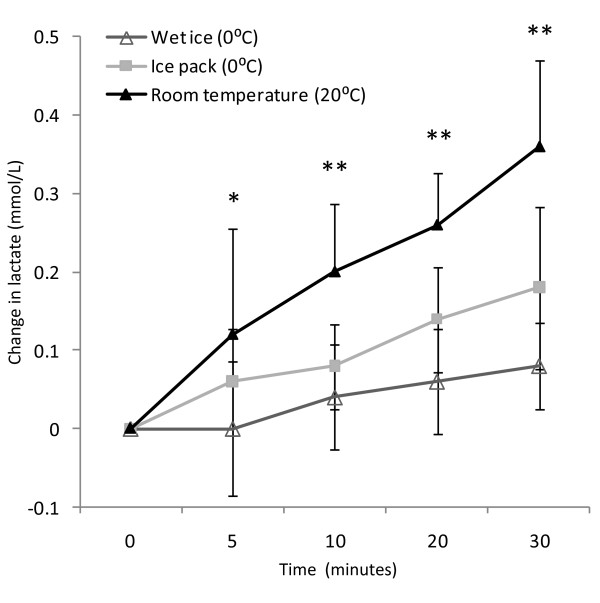
**Change in whole blood lactate over time**. Mean change in whole blood, venous lactate observed from time of initial measurement up to 30 minutes. Error bars represent 95% confidence interval, only upper bar shown for room temperature. * p < 0.05 comparing room temperature to wet ice, ** p < 0.05 comparing both room temperature to wet ice and room temperature to portable ice pack, respectively. *Open triangles *= wet ice (0°C); *grey squares *= portable ice pack (0°C); *black triangles *= room temperature (20°C).

Mean baseline lactate among resting subjects (N = 5) was 1.24 mmol/L (95%CI: 0.49, 1.98 mmol/L). After 30 minutes (Table 1), lactate concentration increased, on average, by 0.08 mmol/L (95%CI: 0.02, 0.13 mmol/L), 0.18 mmol/L (95%CI: 0.07, 0.28 mmol/L), and 0.36 mmol/L (95%CI: 0.24, 0.47 mmol/L) when stored in wet ice, ice pack, and room temperature, respectively. Mean increase in lactate for room temperature was statistically greater compared to wet ice within 5 minutes of measurement (Figure 1). This difference remained both statistically and clinically significant (>0.2 mmol/L) through 30 minutes after sampling. Mean differences between ice pack and room temperature were also statistically significant at 10, 20 and 30 minutes (p < 0.05). Whole blood lactate differences between wet ice and the ice pack were not clinically or statistically significant at any time point (p > 0.05 for all). At 30 minutes, the mean difference in lactate increase between wet ice and ice pack was 0.1 mmol/L (95% CI: 0.018, 0.19), less than the *a priori *threshold for clinical significance.

**Table 1:** Lactate values in mmol/L for each volunteer (labeled A-E) in each temperature and time condition

	0° Celsius (Wet ice)					0° Celsius (Ice pack)					20° Celsius				
**Time (minutes)**	**A**	**B**	**C**	**D**	**E**	**A**	**B**	**C**	**D**	**E**	**A**	**B**	**C**	**D**	**E**

0	1.2	1.1	1.1	0.9	1.9	1.1	1.1	1.2	0.9	2.1	1.4	1	1.2	0.9	1.8
5	1.1	1.1	1.1	1	1.9	1.2	1.2	1.3	0.9	2.1	1.7	1.1	1.3	0.9	1.9
10	1.2	1.1	1.2	1	1.9	1.2	1.2	1.3	1	2.1	1.7	1.2	1.4	1	2
20	1.3	1.1	1.2	1	1.9	1.3	1.3	1.3	1	2.2	1.6	1.3	1.5	1.1	2.1
30	1.3	1.2	1.2	1	1.9	1.3	1.4	1.4	1	2.2	1.8	1.4	1.7	1.1	2.2

## Discussion

We observed that whole blood lactate measurements from specimens wrapped in a portable ice pack were not clinically or statistically different from those stored on ice for up to thirty minutes after sampling. Consistent with prior studies, lactate measurements from whole blood maintained at room temperature were significantly increased beyond samples stored at 0°C. These results inform future studies which seek to measure biomarkers during out-of-hospital care.

The current results confirm that an easy-to-apply, portable ice pack maintained stable lactate concentrations for up to thirty minutes. Cold temperatures are known to slow the reduction of pyruvate to lactate in phlebotomy samples, and the storage of lactate samples prior to analysis in wet ice is advocated as the standard of care. Our experimental system (ice pack at 0°C) prevented clinically-relevant or statistically-significant variability over time, consistent with multiple studies evaluating lactate concentrations after pre-analytic time delays of 30 minutes or longer when maintained on wet ice [8,9]. Although some reports suggest room temperature storage is sufficient for delays less than 15 minutes [10], the current experiment revealed significant differences as early as five minutes using room temperature storage.

### Strengths

Our results have useful implications. Out-of-hospital biomarker measurements have the potential to improve recognition and prognostication for the critically ill in many ways. For example, pre-hospital lactate measurements may improve triage in injured patients [4] or may identify patients who require more intensive therapies than expected based on clinical physiologic measures. Key to these steps is accurate measurement of lactate concentration and rigorous examination of the reliability of testing devices in the field and/or hospital. Our experimental conditions provide a comparator for future studies of handheld measurements. In addition, among EMS systems with transport times up to 30 minutes, our data demonstrate that whole blood samples could be safely transported by EMS using an ice pack at 0°C to hospital laboratories for standard assays. These measurements on pre-hospital samples could directly inform hospital-based, emergency care soon after hospital arrival, and serve as a baseline for hospital-based measurements.

### Limitations

The current study has limitations. First, we evaluated resting subjects, and were unable to determine the trajectory of whole blood lactate measurements obtained during stress or vigorous exercise. We also did not measure platelets, white blood cell, or red blood cell counts, which act as reservoirs for glycolytic enzymes that increase lactate during specimen storage [6]. Therefore, the external validity of our findings to patients with systemic inflammatory response or leukocytosis is unknown, though we suspect the use of healthy subjects lends a conservative bias. As noted previously [10], the majority of patients who present with sepsis have initial lactate concentrations in the normal range. We did not evaluate anti-glycolytic agents, such as sodium fluoride or potassium oxalate [8], as fluoride does not significantly inhibit glycolysis within the time frame for this set of experiments [11]. Although arterial lactate may more accurately reflect tissue hypoperfusion, we did not study arterial samples due to the difficulties in routine arterial puncture during pre-hospital care.

## Conclusions

In summary, we observed that applying a portable ice pack to venous whole blood samples prevented both statistically and clinical significant, pre-measurement increases in lactate concentrations in contrast to samples maintained at room temperature. Future studies of out-of-hospital biomarkers may consider these post-sampling conditions when studying the role of pre-hospital lactate in patients with critical illness.

## Competing interests

The authors declare that they have no competing interests.

## Authors' contributions

CWS, DC, TDR, EMB, and GSB conceived of the study. CWS and GSB designed the study. CWS and GSB performed data collection. CWS, CRC, GSB, and TRW performed data analysis. CWS drafted the manuscript. All authors interpreted the data and performed critical review and contribution to the manuscript. CWS obtained funding for the study. All authors read and approved the final manuscript.
